# Characterization of Influenza A (H7N9) Viruses Isolated from Human Cases Imported into Taiwan

**DOI:** 10.1371/journal.pone.0119792

**Published:** 2015-03-06

**Authors:** Ji-Rong Yang, Chuan-Yi Kuo, Hsiang-Yi Huang, Fu-Ting Wu, Yi-Lung Huang, Chieh-Yu Cheng, Yu-Ting Su, Ho-Sheng Wu, Ming-Tsan Liu

**Affiliations:** 1 Center for Research, Diagnostics and Vaccine Development, Centers for Disease Control, Taipei, Taiwan, ROC; 2 School of Medical Laboratory Science and Biotechnology, Taipei Medical University, Taipei, Taiwan, ROC; Centers for Disease Control and Prevention, UNITED STATES

## Abstract

A novel avian influenza A (H7N9) virus causes severe human infections and was first identified in March 2013 in China. The H7N9 virus has exhibited two epidemiological peaks of infection, occurring in week 15 of 2013 and week 5 of 2014. Taiwan, which is geographically adjacent to China, faces a large risk of being affected by this virus. Through extensive surveillance, launched in April 2013, four laboratory-confirmed H7N9 cases imported from China have been identified in Taiwan. The H7N9 virus isolated from imported case 1 in May 2013 (during the first wave) was found to be closest genetically to a virus from wild birds and differed from the prototype virus, A/Anhui/1/2013, in the MP gene. The other three imported cases were detected in December 2013 and April 2014 (during the second wave). The viruses isolated from cases 2 and 4 were similar in the compositions of their 6 internal genes and distinct from A/Anhui/1/2013 in the PB2 and MP genes, whereas the virus isolated from case 3 exhibited a novel reassortment that has not been identified previously and was different from A/Anhui/1/2013 in the PB2, PA and MP genes. The four imported H7N9 viruses share similar antigenicity with A/Anhui/1/2013, and their HA and NA genes grouped together in their respective phylogenies. In contrast with the HA and NA genes, which exhibited a smaller degree of diversity, the internal genes were heterogeneous and provided potential distinctions between transmission sources in terms of both geography and hosts. It is important to strengthen surveillance of influenza and to share viral genetic data in real-time for reducing the threat of rapid and continuing evolution of H7N9 viruses.

## Introduction

In March 2013, a novel influenza A influenza virus, influenza A (H7N9), that causes severe respiratory infections, with a case fatality rate of 30%, was identified in humans in eastern China [1]. As of May 12, 2014, a total of 437 laboratory-confirmed cases of human infection with the H7N9 virus, including 146 deaths, have been reported [2,3]. Epidemiological data suggests that infections with H7N9 viruses have occurred in two waves: the first wave occurred in February through May, 2013, followed by sporadic cases in July and August (the inter-wave period), and a second wave of human cases has been occurring from October 2013. The number of new cases increased sharply after January 2014, and a peak in new cases that exceeded that of the first wave occurred in February [4]. Most patients have reported previous exposure to birds or live poultry markets [5–7]. The H7N9 virus has also been detected in poultry and environmental samples, suggesting that the virus has been transmitted directly from poultry to humans and that H7N9 virus-infected poultry may be the primary source of human infections [5].

Initial genetic characterization of the human H7N9 viruses isolated earlier in the outbreak has suggested that they were novel reassortants that had emerged from at least four sources; the HA and NA genes were derived from H7 viruses circulating in domestic ducks and N9 viruses circulating in migratory birds, respectively [8,9], and the six internal genes were derived from avian H9N2 viruses that had been circulating recently in China. Comparisons of H7N9 viruses isolated from humans, poultry and environmental samples further revealed limited antigenic diversity [10], and the genes encoding the HA and NA surface proteins were shown to be similar across the viral strains examined with ~2% nucleotide differences [11]. In contrast, the internal genes of these H7N9 viruses were found to have become more diverse, through reassortment with multiple avian influenza A (H9N2) viruses and ~3% nucleotide differences were observed [11]. It has been demonstrated that the six internal genes of the novel H7N9 virus originated from the reassortment of two separate H9N2 strains that have been circulating within Chinese poultry populations for several years and that human H7N9 isolates have evolved into at least two different lineages [6]. Moreover, it has been suggested that the avian influenza A (H9N2) virus may contribute to the evolution of the human H7N9 virus; the coexistence of avian H7N9 and H9N2 viruses in poultry has been linked to divergent viral genome characteristics [12], with a high degree of diversity in the internal genes in particular, as was demonstrated by the finding of 27 distinct genotypes reflecting unique combinations of H9N2 and H7N9 viruses [11,13]. Analyses of viral genomic signatures have shown that some H7N9 viruses possess the Q226L mutation in the HA protein, reflecting a probable change in receptor-binding specificity from avian to mammalian receptors and thus potentially increasing the risk of transmission of the virus among humans [8]. Other genomic substitutions, including E627K in PB2 and R292K in NA, related to mammalian host adaptation and oseltamivir resistance, respectively, have also been reported [5,8].

In Taiwan, four imported H7N9 cases from mainland China have been identified to date. The first patient, a man who returned from Jiangsu Province, China before developing symptoms, was identified in April 2013 during the first H7N9 wave [14]. The patient did not report any history of contact with sick persons or animals during his travels. From December 2013 onward, three additional laboratory-confirmed cases were identified.

Because the genetic and antigenic characteristics of the H7N9 virus during the second wave is limited, in this study, we investigated the genetic features of the novel H7N9 virus by analyzing the complete genome sequences of the four viral isolates and examining evolutionary differences between the viruses from the second epidemic wave and those from the first wave. It was shown that the HA and NA genes from each of the four H7N9 viruses both clustered into the major clade in the respective phylogenetic classification, together with most of the H7N9 viruses isolated from China and Hong Kong. Of note, all four imported viruses were found to belong to minor genotypes, based on the compositions of their six internal genes, and a novel Genotype (G3.6) was identified, as well. These results indicate that H7N9 viruses exhibit genetic heterogeneity and continue to undergo evolution through reassortment. Because human cases of H7N9 infection have been reported continuously, this novel virus has been considered to pose a potential threat to public health; thus, enhanced surveillance is needed.

## Materials and Methods

### Influenza virus isolation and identification

Sputum and/or throat swabs were collected from reported and suspected influenza A (H7N9) cases who exhibited influenza-like illnesses and had traveled to affected provinces or cities in China during the 10 days before the onset of illness [15], and the samples were transported to the laboratories of the influenza surveillance network in Taiwan or to the Taiwan Centers for Disease Control (Taiwan CDC) for diagnosis. Real-time reverse transcription-polymerase chain reaction (RT-PCR) assays were used to determine the influenza types and subtypes of the isolated viruses [16,17]. Influenza A virus isolates that remained unsubtyped by the H1 and H3 assays were further analyzed by primer and probe sets specific for the H5, H6 and H7 subtypes [18–20]. To isolate the influenza A (H7N9) virus, specimens of sputum or throat swabs were inoculated into 9- to 11-day old specific pathogen-free embryonated chicken eggs and incubated for 72 hours at 36°C.

### Genetic analysis of the influenza A (H7N9) virus isolates

To determine viral nucleotide sequences, viral RNA was extracted from suspensions of cultured isolates using QIAamp Viral RNA Mini Kits (Qiagen, Santa Clara, CA, USA), according to the manufacturer’s instructions or in conjunction with an automated extraction system, the MagNA Pure Compact Instrument (Roche, Penzberg City, Germany). Conventional RT-PCR was performed to amplify each genome segment of the H7N9 virus using universal primer sets [21] and the OneStep RT-PCR Kit (Qiagen, Santa Clara, CA, USA). Nucleotide sequences of the amplified PCR products were determined and translated into amino acid sequences. The sequencing reactions were performed bidirectionally on a 3730 DNA Analyzer (Applied Biosystems, Life Technologies, USA). The resulting sequence contigs were assembled using Sequencher version 5.0 sequence analysis software (Gene Codes Corporation, Ann Arbor, MI, USA), and phylogenetic analyses of the complete, assembled viral sequences were performed. Multiple sequence alignments, protein translation and phylogenetic analyses were performed on the basis of the nucleotide sequences using the software MEGA5 [22] and BioEdit (http://www.mbio.ncsu.edu/BioEdit/bioedit.html). Phylogenetic trees were constructed using the Maximum likelihood method, and 1000 bootstrap replications were performed to evaluate the reliability of the trees that were generated. For constructing phylogenetic trees of each gene segment, the available full-length sequences of selected influenza A (H7N9) viruses isolated in China and Hong Kong were downloaded from either the NCBI Influenza Virus Resource [23] or the GISAID (http://platform.gisaid.org) databases and used as references.

### Genotypic classification of the influenza A (H7N9) virus

The genotype of each H7N9 virus was assigned according to the rules of classification for each internal gene, as reported previously [11]. Viruses were assigned to genotype G0, represented by A/Anhui/1/2013, if all internal genes fell into the major clade (Clade 1) on their respective phylogenetic trees. Internal genes of the representative viruses A/duck/Jiangsu/YC2/2013 (H9N2) and A/Hong Kong/5942/2013 (H7N9) were classified as belonging to Clade 2 and Clade 3, respectively. A strain with one internal gene different from G0 in the phylogenetic classification was assigned the genotype G1, and so on, so that a virus with all six internal genes different from the G0 genotype was assigned the G6 genotype. Within each genotype, sub-genotypes were further determined based on the topology of the major clade in the phylogenetic classification.

### Hemagglutination inhibition (HI) assay

Antigenic characteristics of the isolated H7N9 viruses were determined using the HI assay. In the HI assay, ferret antibodies raised against influenza A (H7N9) viruses (A/Anhui/1/2013 and A/Taiwan/1/2013) were used to test antigenic differences between the four viral strains. The virus strain of A/Anhui/1/2013 was kindly provided by the Chinese Center for Disease Control and Prevention (China CDC). The sera were treated with receptor-destroying enzyme (RDE; Denka Seiken, Japan) at a dilution of 1:3 and incubated overnight at 37°C to remove non-specific inhibitors, after which they were heat-inactivated at 56°C for 30 min. RDE-treated sera were further diluted with PBS to a final 1:10 dilution, at which point the sera were ready to be tested. The HI assays were performed as described previously [24]. HI titers were given as the reciprocal of the highest dilution of serum that inhibited virus-induced hemagglutination. Sera that tested negative at a dilution of 1:10 were recorded as a titer of <10.

### Sequence data

The nucleotide sequences of the influenza viruses included in this study have been submitted to the GISAID database. Their accession numbers are EPI1521915–1521917, EPI1521919, EPI1531794–1531805 and EPI541771-EPI541778.

## Results

### Detection of the influenza A (H7N9) virus in Taiwan in 2013 and 2014

**Fig 1:**
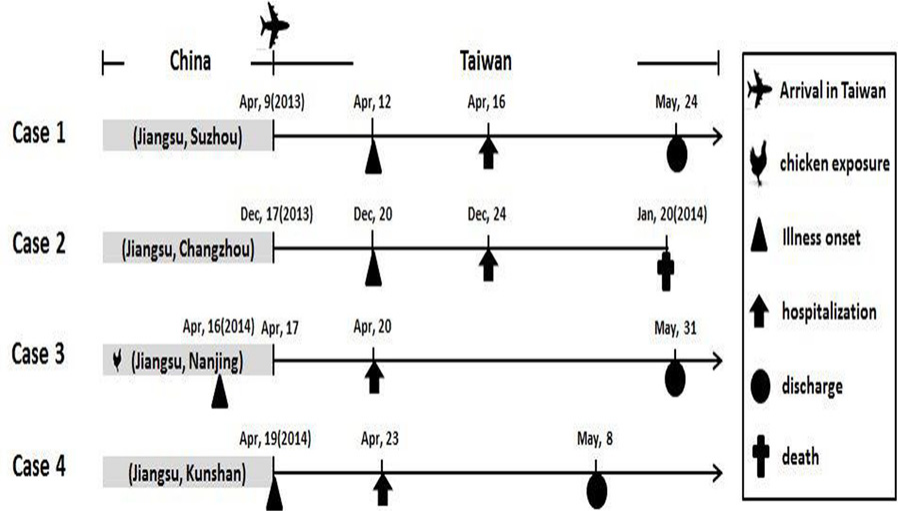
Information about the four confirmed H7N9 cases. The time points (dates) of previous chicken exposure, arrival in Taiwan, illness onset hospitalization and discharge for each case are indicated by symbols, as shown in the key. Provinces and cities where each imported case carried out daily activities before arrival in Taiwan are indicated in gray shadow boxes.

**Fig 2:**
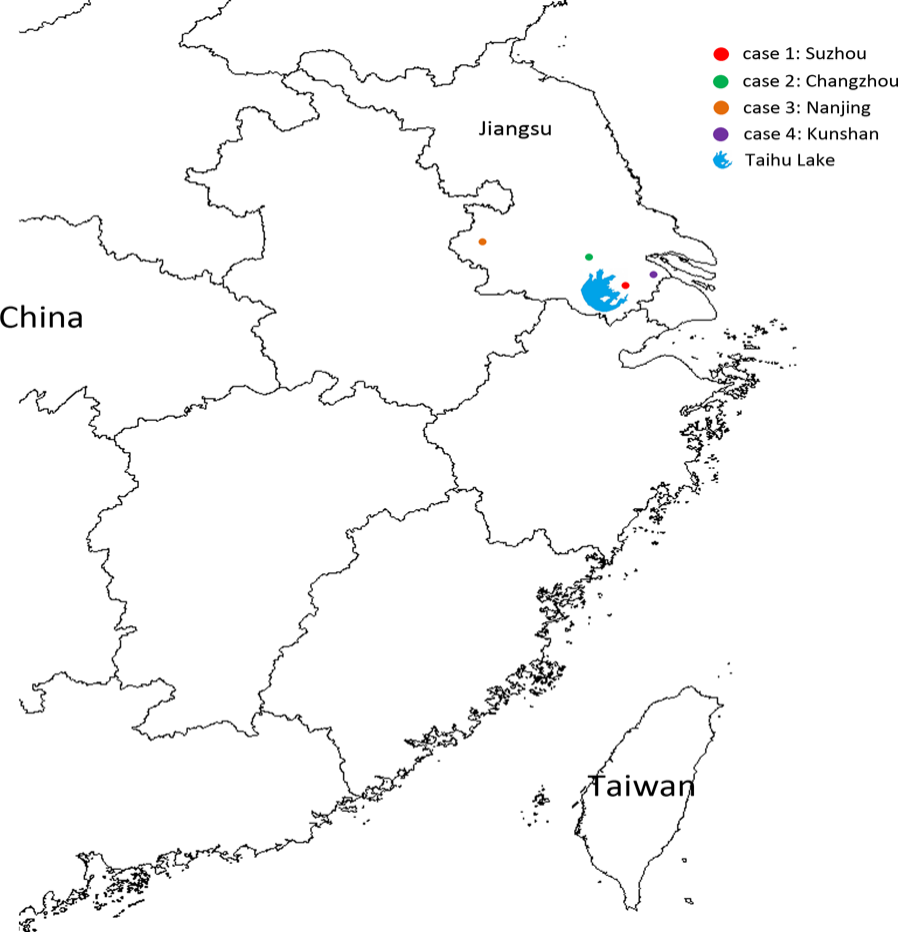
Geographical locations in mainland China of the four laboratory-confirmed cases of influenza A (H7N9) before arrival in Taiwan. The locations of the cases are represented by dots, with a different color for each case. The location of Taihu Lake is marked with a symbol, as indicated.

From April 3, 2013 to May 12, 2014, a total of 596 patients (531 Taiwanese and 65 Chinese) who developed influenza-like illness and had histories of travel to China before the onset of symptoms were reported to the Taiwan CDC. Sputum and/or swab specimens were collected from these patients, and diagnostic tests for infection with the H7N9 virus were conducted. Among these patients, four imported H7N9 cases from mainland China were confirmed by real-time RT-PCR and isolation of the H7N9 virus. Of the 592 H7N9-negative patients, 88 cases tested positive for influenza A (H1N1)pdm09, 58 for influenza A (H3N2) and 15 for influenza B viruses. Information about the four confirmed H7N9 cases, including clinical histories and regions in which each patient lived or traveled before the onset of illness, are reviewed in Fig. 1. Detailed comparison of clinical information among the 4 H7N9, 88 H1N1pdm09, 58 H3N2 and 15 influenza B cases identified through the enhanced influenza surveillance was shown in Table 1. All the four H7N9 cases had severe illness with pneumonia and hospitalized in intensive care unit; one patient died. Notably, among the four imported H7N9 cases reported in this study, only the case 3 patient had a history of contact with chickens, in the form of contact with slaughtered chickens in the wet market 11 days before the onset of his symptoms. The cases 1, 2 and 4 patients did not reported any history of exposure to poultry, including the visits to live poultry markets or any other forms of animal markets, farms or zoos. All four cases had daily activities in cities in Jiangsu Province, including Suzhou (case 1), Changzhou (case 2), Nanjing (case 3) and Kunshan (case 4), before arrival in Taiwan; these cities are all located near Taihu Lake, the third largest freshwater lake in China (Fig. 2). All four cases had to be admitted to the hospital 4 days after the onset of symptoms.

**Table 1:** Comparison of clinical information between the H7N9- and seasonal influenza virus-infected patients confirmed in an enhanced influenza surveillance of H7N9 in Taiwan, from April 3, 2013 to May 12, 2014.

Clinical information	No. (%) of the virus-infected patients			
	H7N9 (n = 4)	H1N1pdm09 (n = 88)	H3N2 (n = 58)	Influenza B (n = 15)
**Clinical condition**				
Fever (≥38°C)	3 (75.0)	81 (92.0)	57 (98.3)	15 (100.0)
Cough	2 (50.0)	66 (75.0)	45 (77.6)	8 (53.3)
Pneumonia	4 (100.0)	6 (6.8)	11 (19.0)	5 (33.3)
Hospitalized	4 (100.0)	39 (44.3)	22 (38.0)	6 (40.0)
Intensive Care Unit (ICU) admission	4 (100.0)	3 (3.4)	0 (0)	0 (0)
**Clinical outcome**				
Death	1 (25.0)	1 (1.1)	0 (0)	1 (6.7)

### Phylogenetic, genotypic and antigenic characteristics of the four H7N9 viruses

**Fig 3:**
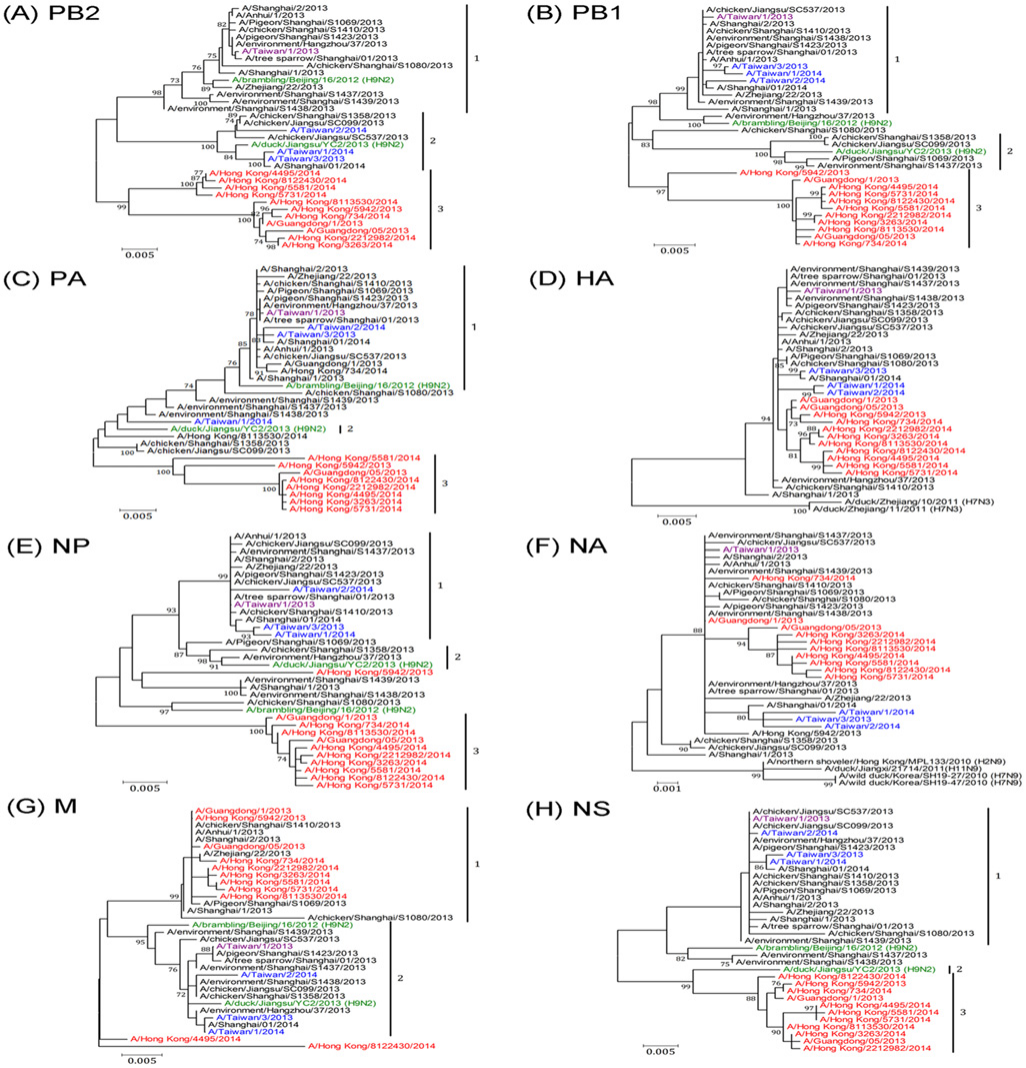
Phylogenetic relationships for (A) PB2, (B) PB1, (C) PA, (D) HA, (E) NP, (F) NA, (G) MP and (H) NS of the four imported H7N9 viruses identified in this study. The phylogenetic trees were constructed using the Maximum likelihood method with 1000 bootstrap replications. Branch values of more than 70 are indicated. Genome sequences of H7N9 viruses isolated from humans, poultry and the environment during the two epidemic waves in China were obtained from the NCBI and GISAID databases and were used as references. The classification of specific evolutionary clades, including the clades 1, 2 and 3, is indicated. The four imported viruses of Taiwan, which were isolated during the first or second waves of the H7N9 epidemic, were indicated by purple and blue colors, respectively. Viruses indicated by the green colors were the H9N2 lineages with genetic constellation related to the H7N9 viruses. Viruses indicated by the red colors were H7N9 strains isolated from Hong Kong and Guangdong in China during the second wave of the H7N9 epidemic.

To investigate the phylogenetic and genetic characteristics of the four imported H7N9 viruses of Taiwan, which were isolated in the period spanning the two H7N9 epidemic waves, we analyzed their complete genome sequences and compared these with several selected, representative genome sets available in the NCBI and GISAID databases. The nucleotide identity of the four imported influenza A(H7N9) and selected representative isolates, compared to the full-length sequences of A/Anhui/1/2013 virus was illustrated in S1 Table. Based on the analyzed phylogenies of the internal genes, the four H7N9 viruses could be classified into more than one group. The PB2 gene of A/Taiwan/1/2013 (isolated from imported case 1 and designated as TW01/13) was located in Clade 1 together with the PB2 gene of the prototype virus A/Anhui/1/2013 and was closely related to that of the H9N2 virus A/brambling/Beijing/16/2012. The PB2 genes of the other three viruses, A/Taiwan/3/2013 (TW03/13, from case 2), A/Taiwan/1/2014 (TW01/14, from case 3), and A/Taiwan/2/2014 (TW02/14, from case 4), were located in Clade 2 along with the H9N2 virus A/duck/Jiangsu/YC2/2013 and could be separated from those of viruses isolated in Hong Kong and Guangdong during the second H7N9 epidemic wave, which viruses typically clustered into Clade 3 (Fig. 3A). The PB1, NP, and NS genes of the four H7N9 viruses fell within Clade 1 together with the majority of human H7N9 isolates from the first epidemic wave (Fig. 3B, 3E and 3H). PA genes from the TW01/13, TW03/13 and TW02/14 viruses were located in Clade 1, while that of the TW01/14 virus fell into Clade 2 along with the H9N2 virus A/duck/Jiangsu/YC2/2013 (Fig. 3C). The MP genes of the four H7N9 viruses belonged to Clade 2 and were thus separated from most of the earlier human isolates from the first H7N9 wave, including A/Anhui/1/2013, and from viruses isolated in Hong Kong and Guangdong in the second wave (Fig. 3G). The surface protein genes, the HA and NA genes, of the four H7N9 viruses clustered together in each respective phylogeny in the major A/Anhui/1/2013-like clade together with most of the representative viruses (Fig. 3D and 3F). Of note, the NA genes of the TW03/13, TW/01/14 and TW02/14 viruses, which belong to the second epidemic wave, formed a sub-cluster in their respective phylogenetic classifications (Fig. 3F). These results suggested that internal genes of the H7N9 viruses in the second wave have exhibited greater diversity and heterogeneity than did the surface protein genes. Mismatches between the tree topologies also mirrored the fact that H7N9 viruses were continuously evolving through reassortment.

Based on the constellation of six internal genes present in each of the four imported H7N9 viruses, these viruses could be classified into three genotypes: the genotype G1.1 (TW01/13), G2.9 (TW03/13 and TW02/14) and G3.6 (TW01/14) (Table 2). The genotype G3.6 was a new reassortant identified in this study, and its constellation of internal genes has not been identified previously. This finding of a new genotype may indicate that the TW01/14 virus originated from reassortment events involving the A/duck/Jiangsu/YC2/2013 (H9N2) virus in the field. Furthermore, none of the four H7N9 viruses belonged to the major genotype G0, which was the most frequently detected genotype in human infections in China [11]. Antigenic characteristics of the viruses were analyzed and compared by HI assay (Table 3). The results of the HI assays shows that all four H7N9 viruses are antigenically similar to the vaccine candidate strain A/Anhui/1/2013 virus, consistent with the finding that their HA sequences are also closely related to each other (Fig. 3 and S1 Table).

**Table 2:** Genotyping of the four imported H7N9 viruses of Taiwan.

Viruses	Host	PB2	PB1	PA	HA	NP	NA	MP	NS	Genotype*
A/Anhui/1/2013	Human	1	1	1	1	1	1	1	1	0
A/pigeon/Shanghai/S1423/2013	Poultry	1	1	1	1	1	1	2	1	1.1
A/chicken/Jiangsu/SC537/2013	Poultry	2	1	1	1	1	1	2	1	2.9
A/Taiwan/1/2013	Human	1	1	1	1	1	1	2	1	1.1
A/Taiwan/3/2013	Human	2	1	1	1	1	1	2	1	2.9
A/Taiwan/1/2014	Human	2	1	2	1	1	1	2	1	3.6
A/Taiwan/2/2014	Human	2	1	1	1	1	1	2	1	2.9
A/Shanghai/1/2014	Human	2	1	1	1	1	1	2	1	2.9
A/Guangdong/1/2013	Human	3	3	1	1	3	1	1	3	4.5
A/Guangdong/5/2013	Human	3	3	3	1	3	1	1	3	5.4
A/Hong Kong/734/2014	Human	3	3	1	1	3	1	1	3	4.5
A/Hong Kong/3263/2014	Human	3	3	3	1	3	1	1	3	5.4

*The classification of the viral genotype was based on the definition reported previously [11] as described in the Materials and Method section of the main text.

**Table 3:** Antigenic analysis of the four imported H7N9 viruses by hemagglutination inhibition assay.

Viruses	Post-infection ferret sera		Genotype	Passagehistory
	A/Anhui/1/2013	A/Taiwan/1/20013		
A/Anhui/1/2013	**320**	320	G0	E2
A/Taiwan/1/2013	320	**640**	G1.1	E3
A/Taiwan/3/2013	320	320	G2.9	E2
A/Taiwan/1/2014	320	160	G3.6	E1
A/Taiwan/2/2014	320	320	G2.9	E1

### Amino acid characteristics in the proteins of the four H7N9 viruses

Specific amino acid substitutions in the viral proteins are listed and compared in Table 4. Amino acid substitutions related to mammalian host adaptation, receptor binding and/or antiviral resistance are listed for the four H7N9 viruses. The Q226L substitution in the HA gene, contributing to high-affinity binding of viruses to human receptors present in the upper respiratory tract, was detected in the TW03/13, TW01/14 and TW02/14 viruses, while TW01/13 had a Q226P substitution, which is uncommon. The E627K substitution in the PB2 protein, which is associated with enhanced polymerase activity, was found in the TW01/13, TW03/13 and TW01/14 viruses; in contrast, the TW02/14 virus had 627E together with the D701N substitution, which has been suggested to compensate for a lack of 627K to increase transmission in mammals [25]. We examined signatures related to antiviral drug susceptibility, including the R292K substitution; this substitution was present in the TW01/13 virus, suggesting that this virus had developed resistance to oseltamivir. All four viruses had the S31N substitution in the M2 protein; this substitution confers resistance to adamantanes.

**Table 4:** Amino acid substitutions, associated with mammalian host adaptation, receptor binding and antiviral resistance, in the proteins of the four imported H7N9 viruses.

Virus	Collection date	PB2		PB1-F2	HA		NA*		M2	NS1
	(mm/dd/yy)	627	701	66	226	228	119	292	31	92
A/Anhui/1/2013	03/20/2013	K	D	N	L	G	E	R	N	D
A/Taiwan/1/2013	04/24/2013	K	D	N	P	G	E	R/K^#^	N	D
A/Taiwan/3/2013	12/27/2013	K	D	N	L	G	E	R	N	D
A/Taiwan/1/2014	04/21/2014	K	D	N	L	G	E	R	N	D
A/Taiwan/2/2014	04/24/2014	E	N	N	L	G	E	R	N	D
Substitutions		E627K	D701N	N66S	Q226L	G228S	E119V	R292K	S31N	D92E

^#^Mixed amino acid residues were detected.

## Discussion

In response to the outbreak of avian influenza A (H7N9) virus in humans in 2013, the Taiwan Centers for Disease Control strengthened influenza surveillance and listed H7N9 infection as a national category V notifiable infectious disease on April 3, 2013 [15]. Any suspected case of H7N9 infection or unexplained pneumonia must be reported, and respiratory specimens must be collected and tested for the presence of the H7N9 virus. Due to this enhanced surveillance, we have found a total of 4 H7N9 cases, all imported from China in May 2013, December 2013 and April 2014. Malaysia has also confirmed an imported human H7N9 case, in February 2014 [3]. Cases imported from China may sporadically be detected in other countries, as well, due to frequent human travel. In China, the virus has also spread widely from the most affected areas, including Shanghai City and Jiangsu, Zhejiang and Anhui Provinces in the first wave, to Guangxi and Guangdong Provinces and Hong Kong in the second epidemic wave [2]. The geographic spread of the virus will increase the risk of human infection and has the potential to cause an influenza pandemic, which possibility is of significant global concern.

Genetic analyses of human H7N9 virus isolates has revealed that this virus is derived from multiple and sequential reassortments between H7N3, HxN9 and H9N2 viruses [8,26,27]. The emergence of more new genotypes with novel constellations of internal gene segments indicates that the virus continues to evolve through reassortment with several H9N2 lineages [28,29]. Aside from the HA and NA genes that formed a large cluster and separated from possible ancestors (Fig. 3) [26], the six internal genes derived from distinct H9N2 lineages can exhibit unique genetic characteristics that can be used to track the geography and hosts of transmission sources. For example, the genotype G0 is the prototype virus that has been predominantly detected in infected humans and poultry and that has been found in several geographical regions, whereas genotypes G4-G6 have been isolated only from poultry and the environment; genotype G6 has been isolated mainly in Zhejiang [11]. The four imported viruses identified in this study were classified as having the minor genotypes G1.1 (TW01/13), G2.9 (TW03/13 and TW02/14) and G3.6 (TW01/14), and none of these belonged to the predominant genotype G0. To explain why these viruses imported into Taiwan were not of the most common genotypes, such as have been observed in the main areas of human H7N9 infection in China, two possibilities are proposed. First, these patients may have become infected from a transmission source other than poultry, which is considered to be potentially the main transmission route for major genotype G0 viruses [5–7]. During the first wave of the H7N9 epidemic in China, contact with poultry, especially in live poultry markets, was regarded as a major source of human H7N9 virus infections because closure of live poultry markets in the affected cities resulted in a decrease in human H7N9 cases [30]. In this study, epidemiologic investigations and viral genetic analyses revealed that three imported cases (cases 1, 2 and 4) had no poultry-related contact history. The TW01/13 virus, isolated in May 2013 (during first wave) from imported case 1, who had travelled to Suzhou, Jiangsu and had not had contact with poultry, was found to be closest genetically to the H7N9 virus of A/tree sparrow/Shanghai/01/2013 (Fig. 3), which was isolated from a wild bird in Shanghai, China [31]. The TW03/13 and TW02/14 viruses isolated from imported cases 2 and 4, who had travelled to Changzhou and Kunchan, Jiangsu, respectively, and who had reported no contact with poultry, were both classified as being of Genotype 2.9, which has previously been found only in chickens, based on the sequences available in databases, and were closest genetically to A/chicken/Jiangsu/SC537/2013, which was isolated in Jiangsu Province. Together, these findings suggest that the transmission sources for cases 1, 2, and 4 should not be assumed to be poultry and that an additional source, such as wild birds, may play a role in the transmission of H7N9 viruses to humans, similar to the role they played in the geographic spread of avian influenza H5N1 viruses [32].

The other possibility may be that the predominant genotype of H7N9 viruses in Jiangsu has changed in the second wave. However, this hypothesis can only be evaluated when sequence data from Jiangsu isolates from the second wave become available. Most sequence data currently available in databases from H7N9 viruses isolated in the second epidemic wave have been from viruses isolated in Guangdong and Hong Kong. Analyses of these sequences have found unique reassortment patterns with local H9N2 viruses and gene constellations that are extensively different from those of viruses isolated in the first wave [28,29]. In this study, the TW03/13, TW01/14 and TW02/14 viruses were isolated from Jiangsu, China in the second wave. Analyses of their complete genome sequences revealed that these viruses were not only distinct from the G0 viruses in the first wave but that their five internal genes (PB1, PB2, NP, MP and NS) were also phylogenetically divergent from reassortants isolated in Hong Kong and Guangdong isolated during the second wave (Fig. 3). This suggests that recruitment of the internal genes of H7N9 viruses from H9N2 viruses may be more complicated than previously thought and that the H9N2 viruses circulating locally in each geographic region likely contribute to the evolution of the H7N9 virus.

In summary, the continuing evolution of H7N9 viruses through reassortment with a variety of H9N2 lineages may lead to rapid increases in viral genetic diversity [11,28,29]. Thus, it is essential to continue to strengthen influenza surveillance and the real-time sharing of viral genetic data, to facilitate risk assessment and the control of H7N9 outbreaks.

## Supporting Information

S1 Table The nucleotide identity of the four imported influenza A(H7N9) and selected representative isolates, compared to the full-length sequences of A/Anhui/1/2013 virus. (PDF)
