# Supplementary material for: Characterization of Influenza A (H7N9) Viruses Isolated from Human Cases Imported into Taiwan
Source: PLoS One. 2015 Mar 6;10(3):e0119792. doi: 10.1371/journal.pone.0119792 (PMC4351886; doi:10.1371/journal.pone.0119792)
Supplement: S1 Table — (PDF) [file pone.0119792.s001.pdf]

Table S1. The nucleotide identity of the four imported influenza A(H7N9) and selected representative isolates, compared to the full-length sequences of A/Anhui/1/2013 virus.

Viruses	Gene segments							
	PB2	PB1	PA	HA	NP	NA	MP	NS
A/Taiwan/1/2013	0.999	0.998	0.998	0.997	1	0.998	0.978	1
A/Taiwan/3/2013	0.966	0.996	0.997	0.996	0.996	0.994	0.976	0.995
A/Taiwan/1/2014	0.965	0.994	0.975	0.994	0.995	0.992	0.977	0.997
A/Taiwan/2/2014	0.962	0.994	0.993	0.994	0.993	0.993	0.976	0.998
A/Shanghai/01/2014	0.965	0.998	0.997	0.996	0.998	0.996	0.977	0.996
A/chicken/Jiangsu/SC537/2013	0.969	0.999	0.999	0.998	1	0.997	0.981	1
A/Guangdong/1/2013	0.963	0.971	0.996	0.997	0.965	0.999	1	0.967
A/Guangdong/05/2013	0.959	0.97	0.962	0.997	0.962	0.995	0.998	0.963
A/Hong Kong/734/2014	0.96	0.969	0.996	0.992	0.963	0.997	0.996	0.965
A/Hong Kong/3263/2014	0.96	0.97	0.964	0.994	0.961	0.994	0.996	0.964
